# Irinotecan plus raltitrexed *vs* raltitrexed alone in patients with gemcitabine-pretreated advanced pancreatic adenocarcinoma

**DOI:** 10.1038/sj.bjc.6600883

**Published:** 2003-04-15

**Authors:** H Ulrich-Pur, M Raderer, G Verena Kornek, B Schüll, K Schmid, K Haider, W Kwasny, D Depisch, B Schneeweiss, F Lang, W Scheithauer

**Affiliations:** 1 Division of Clinical Oncology, Department of Internal Medicine I, University Hospital, Waehringer Guertel 18-20, A-1090 Vienna, Austria; 2 Department of Surgery, Wr.Neustadt General Hospital, Corvinusring 3-5, A-2700 Wr.Neustadt, Austria; 3 Department of Internal Medicine, Kirchdorf General Hospital, Hausmanningerstrasse 8, A-4560 Kirchdorf a.d.Krems, Austria; 4 Department of Surgery, Neunkirchen General Hospital, Peischingerstrasse 19, A.2024 Neunkirchen, Austria

**Keywords:** pancreatic cancer, chemotherapy, second-line treatment, irinotecan, raltitrexed

## Abstract

There is no established second-line treatment for advanced pancreatic cancer after gemcitabine failure. In view of the urgent need for such therapy, and since preclinical and phase I clinical data suggest an encouraging, potentially synergistic activity between raltitrexed and irinotecan, the present randomised phase II study was initiated. A total of 38 patients with metastatic pancreatic adenocarcinoma, who progressed while receiving or within 6 months after discontinuation of palliative first-line chemotherapy with gemcitabine, were enrolled in this study. They were randomised to 3-weekly courses of raltitrexed 3 mg m^−2^ on day 1 (arm A) or irinotecan 200 mg m^−2^ on day 1 plus raltitrexed 3 mg m^−2^ on day 2 (arm B). The primary study end point was objective response, secondary end points included progression-free survival (PFS) and overall survival (OS), as well as clinical benefit response in symptomatic patients (*n*=28). In the combination arm, the IRC-confirmed objective response rate was 16% (three out of 19 patients had a partial remission; 95% CI, 3–40%), which was clearly superior to that in the comparator/control arm with raltitrexed alone, in which no response was obtained. Therefore, the trial was already stopped at the first stage of accrual. Also, the secondary study end points, median PFS (2.5 *vs* 4.0 months), OS (4.3 *vs* 6.5 months), and clinical benefit response (8 *vs* 29%) were superior in the combination arm. The objective and subjective benefits of raltitrexed+irinotecan were not negated by severe, clinically relevant treatment-related toxicities: gastrointestinal symptoms (42 *vs* 68%), partial alopecia (0 *vs* 42%), and cholinergic syndrome (0 *vs* 21%) were more commonly noted in arm B; however, grade 3 adverse events occurred in only three patients in both treatment groups. Our data indicate that combined raltitrexed+irinotecan seems to be an effective salvage regimen in patients with gemcitabine-pretreated pancreatic cancer. The superior response activity, PFS and OS (when compared to raltitrexed), as well as its tolerability and ease of administration suggest that future trials with this combination are warranted.

Pancreatic adenocarcinoma, which is responsible for approximately 5% of all cancer-related deaths in the Western world (Parker *et al*, 1996), continues to be a major unresolved health-care problem. The large majority of patients present with disease that is beyond the scope of surgical cure, and their clinical course is characterised by debilitating symptoms and an extremely poor prognosis (Schnall *et al*, 1996). Although it has been reported that 5-fluorouracil (FU)-based chemotherapy is superior than best supportive care alone (Palmer *et al*, 1994; Glimelius *et al*, 1996), and that gemcitabine even offers a survival advantage over FU combined with an increased level of palliation as evidenced by clinical end points of pain intensity, analgesic use, and performance status (Burris *et al*, 1997), overall therapeutic results are still disappointing: in the latter study, apart from the fact that FU was used in a suboptimal way, that is, a single weekly short-term infusion rather than a continuous infusional schedule with or without leucovorin, the reported objective response rate in the ‘winner arm’ was only 5%. Similarly, there was only a modest survival advantage (5.6 *vs* 4.4 months), and only one out of four patients (24%) experienced clinical benefit (Burris *et al*, 1997). Therefore, a number of clinical research efforts are currently being undertaken in order to enhance the therapeutic effectiveness of front-line chemotherapy in advanced disease, including modification of the administration schedule of gemcitabine (Temporo *et al*, 1999), dose intensification (Ulrich-Pur *et al*, 2000), and/or its combination with other drugs and biologicalsQ1 (Heinemann, 2002). Despite encouraging phase I/II study results, however, definitive superior treatment results are still eagerly awaited.

An additional problem in the therapeutic management of this common malignant disease constitutes the need for effective treatment alternatives in patients failing gemcitabine-based chemotherapy. Based on previous data suggesting some activity of the topoisomerase I inhibitor irinotecan in this treatment setting (Klapdor and Fenner, 2000), Q2and *in vitro* (Aschele *et al*, 1998) and *in vivo* evidence (Ford *et al*, 2000; Stevenson *et al*, 2001) of a schedule-dependent synergy with the quinazoline antifolate raltitrexed, a potent and selective inhibitor of thymidilate synthase (Van Cutsem *et al*, 2002), the present study was undertaken. The aim of this randomised multicentre phase II study was to investigate the feasibility and therapeutic index of combined irinotecan plus raltitrexed *vs* raltitrexed alone in patients with advanced pancreatic adenocarcinoma, who had failed or recurred after prior palliative gemcitabine-based first-line chemotherapy.

## PATIENTS AND METHODS

### Patients selection

Patients with histologically confirmed metastatic pancreatic adenocarcinoma and bidimensionally measurable disease (defined as presence of at least one index lesion capable of two-dimensional measurement by computed tomography (CT) scan outside any irradiated zone and ⩾2 cm in diameter) were considered candidates for this study. All patients must have developed progressive disease (PD) while receiving or within 6 months after discontinuing palliative gemcitabine-based chemotherapy. An interval of at least 4 weeks with full resolution of all toxicities had to elapse before administration of the study medication. Eligibility criteria also included a Karnofsky performance index of at least 50%, age between 19 and 75 years, adequate bone marrow reserve (leukocyte count ⩾4000 *μ*l^−1^, platelet count ⩾100 000 *μ*l^−1^), adequate renal function (serum creatinine concentration <132 *μ*mol), and adequate hepatic function (serum bilirubin level <34 *μ*mol l^−1^ and serum transaminase level < two times the upper limits of normal). Patients may not have received more than one palliative gemcitabine-based chemotherapy regimen and/or extensive prior radiation therapy of more than 20% of the bone marrow. Patients who received radiation therapy with a target lesion outside the radiation port were allowed to participate. Similarly, adjuvant chemo- and/or radiotherapy were acceptable. Exclusion criteria included the presence of CNS metastases, serious or uncontrolled concurrent medical illness, and a history of other malignancies, with the exception of excised cervical or basal skin/squamous cell carcinoma. Informed consent according to institutional regulations was obtained from all patients before study entry.

## RANDOMISATION PROCEDURES

Before randomisation, patient eligibility was confirmed by a protocol-specific check list. After signing informed consent documents, and a 2–7-day pain stabilisation lead-in period in symptomatic patients (that aimed to provide adequate pain control and to establish base-line measures for clinical benefit response assessment), patients were stratified according to Karnofsky performance score (90–100 *vs* 50–80%), and prior response to gemcitabine first-line chemotherapy (Complete remission (CR), partial remission (PR), stable disease (SD) *vs* progression). Patients were then assigned to one treatment regimen by the central office located at the University in Vienna.

### Treatment protocol

In both treatment arms, an identical conventional dose regimen of raltitrexed (3 mg m^−2^ given as a 15-min intravenous (i.v.) infusion on day 1) was used. In the combination arm B, according to the described schedule-dependent synergy (Aschele *et al*, 1998), the thymidilate synthase inhibitor was given on day 2, 24 h after irinotecan. Based on the results of a small disease-oriented phase I investigation in patients with advanced pancreatic cancer (unpublished data), a lower dose of the latter drug (200 mg m^−2^) was administered as recommended (350 mg m^−2^) in two previously published phase I studies in other solid tumours (Ford *et al*, 2000; Stevenson *et al*, 2001). In both treatment arms, courses were repeated every 3 weeks for a total of six courses unless there was prior evidence of PD. Concomitant medications routinely given before cytotoxic drug administration included ondansetron 8 mg (plus dexamethasone 8 mg in patients randomised to arm B). If severe cholinergic symptoms were observed during or after irinotecan infusion, 0.25 mg of atropine given as a subcutaneous injection was recommended and prophylactically administered during subsequent courses. Specific guidelines for treatment of delayed diarrhoea were provided, which recommended 2 mg of loperamide every 2 h until more than 12 h had passed after the last loose stool.

### Toxicity and dosage modification guidelines

Adverse reactions were evaluated according to World Health Organization (WHO) criteria (Miller *et al*, 1981). Chemotherapeutic drug doses were reduced by 25% in case of grade 4 haematotoxicity and/or if any other severe (⩾WHO grade 3) organ toxicity was observed in the previous cycle. Treatment could be delayed for up to 2 weeks until adverse effects resolved or at least improved to grade 1. Any patient who required more than 2 weeks for recovery of adverse reactions was taken off the study.

### Pretreatment and follow-up evaluation

Before chemotherapy was initiated, all patients were assessed by physical examination, routine haematology and biochemistry analyses, chest X-ray, and CT scans to define the extent of the disease. Complete blood cell counts with platelet and differential counts were obtained weekly during chemotherapy, and serum chemistry analyses were repeated at least once every course. Subjective symptoms, physical examination results, adverse reactions, performance status, and other clinical benefit response parameters were recorded before each treatment cycle. Objective tumour reassessments were performed every 2 months.

### Study objectives

The primary efficacy end point was objective response rate, which was assessed every 2 months using WHO standard criteria. In case of PR or CR, a second assessment 4 weeks later was required for confirmation of response. All tumour measurements were reviewed and confirmed by an independent panel of oncologists and radiologists (IRC).

Secondary efficacy end points included PFS and OS time, as well as clinical benefit response in symptomatic patients, which was evaluated as previously described (Burris *et al*, 1997).

### Statistical methods

The Simon (1989) two-stage Q3optimal design was used to determine the number of patients in this phase II study. With a 5% alpha risk and a 15% beta risk, we determined a first-stage response probability of 5% (which if true, implied discontinuing the trial) and a minimal rate of efficacy of 15% (which if true, implied moving on to the second stage of the trial). The number of patients to be included in each arm was calculated to be 19 for the first stage and an additional 35 for the second stage. After the inclusion of 54 patients in each arm, the observation of four or fewer patients with objective responses allowed a conclusion of insufficient treatment efficacy. Differences in distribution of patients between the two arms of the trial were Q4evaluated with a *χ*^2^ test (Cochran, 1954). The exact binominal confidence interval was applied to estimate the response rates. Progression-free survival and OS were examined with the Kaplan–Meier product-limit method (Kaplan and Meier, 1958).

## RESULTS

### Patients' characteristics

Between July 2000 and September 2001, a total of 38 patients were entered onto this trial from four different institutions: 19 patients on both treatment arms, all of whom were considered evaluable for response and toxicity assessment. The trial was already stopped at the end of the first stage of accrual because of the response rates achieved. Table 1

**Table 1:** Patients' characteristics

	**Raltitrexed**	**Raltitrexed+irinotecan**
Number of patients	19	19
		
*Gender*		
Male	8	12
Female	11	7
		
Median age in years (range)	60 (40–74)	63 (49–75)
		
*Karnofsky performance status (%)*		
90–100	4 (21%)	4 (21%)
70–80	10 (53%)	9 (47%)
50–60	5 (26%)	6 (32%)
		
*Prior surgery*		
Whipple	5 (26%)	4 (21%)
Palliative bypass	4 (21%)	6 (32%)
Stent	5 (26%)	5 (26%)
None	5 (26%)	4 (21%)
		
*Response to first-line chemotherapy*^a^		
PR	2 (11%)	3 (16%)
SD	9 (47%)	8 (42%)
PD	8 (42%)	8 (42%)
		
*Sites of metastases*		
Abdominal mass	15 (79%)	16 (84%)
Liver	14 (74%)	12 (63%)
Lung	5 (26%)	4 (21%)
Spleen	1 (5%)	2 (11%)
Adrenals	1 (5%)	1 (5%)
Soft tissue	2 (11%)	3 (16%)
		
*Histological grading*		
1	2 (11%)	1 (5%)
2	10 (53%)	12 (63%)
3	7 (37%)	6 (32%)

a All patients had received palliative first-line treatment with bimonthly high-dose gemcitabine. PR=partial remission, SD=stable disease, PD=progressive disease.

lists demographic data, baseline disease characteristics, and prior therapeutic interventions for all randomised patients. Apart from a higher proportion of female subjects in the raltitrexed arm, the two groups were well balanced. Most patients were elderly, and the large majority had multiple intra-abdominal sites of metastases. In both groups, 21–26% of patients had undergone prior potential curative surgery with disease recurrence after a median of 6–8 months. Four (arm A) *vs* six patients (arm B) had undergone palliative surgery for biliary and/or gastric decompression, and five patients each had received endoscopic stents for relieving obstructive jaundice before study entry. Palliative first-line treatment with gemcitabine monotherapy was effected in all patients in both treatment arms, and has resulted in abrogation of PD (PR, SD) in 58%. A total of 12 patients (63%) in the raltitrexed group, and 14 (74%) in the combination arm were suffering from disease-related symptoms at study entry and were considered evaluable for clinical benefit response. In most of these patients, pain was the predominant symptom: 11 (92%) on raltitrexed and 12 (86%) on raltitrexed+irinotecan had a baseline pain intensity score greater than 20 points, and more than 90% in each group required more than 10 morphine-equivalent mg day^−1^ for control of pain. Similarly, most patients had an impaired performance status at study entry. A Karnofsky performance score of 50–80% was recorded in 15 patients (79%) in both treatment arms. More than two-thirds of the patients had experienced weight loss, ranging from 10 to 43% of premorbid body weight.

### Treatment summary

Of the 38 patients enrolled, all received at least one dose of the allocated treatment, almost two-thirds completed 12 weeks of therapy, and 26% (three in arm A and seven in arm B) completed the planned treatment period of 24 weeks. Treatment was stopped early in only one patient in the raltitrexed + irinotecan combination arm for adverse reactions; in all other patients the reason for treatment discontinuation was PD. Both treatment groups adhered closely to the planned dosage regimens. For patients treated with raltitrexed alone, the mean duration of treatment was 11 weeks, and for those treated with raltitrexed+ irinotecan, the mean duration of treatment was 14 weeks.

### Antitumour efficacy

According to the IRC assessment, there was no objective response in arm A, and three responses occurred in arm B for a total response rate of 16% (95% CI, 3–40%). All responses in the combination arm were partial, occurred within 3 months of therapy, and lasted for a duration of 4, 5, and 7.5 months, respectively. The study was therefore closed, and arm B was declared ‘the winner’ according to our study design.

**Figure 1:**
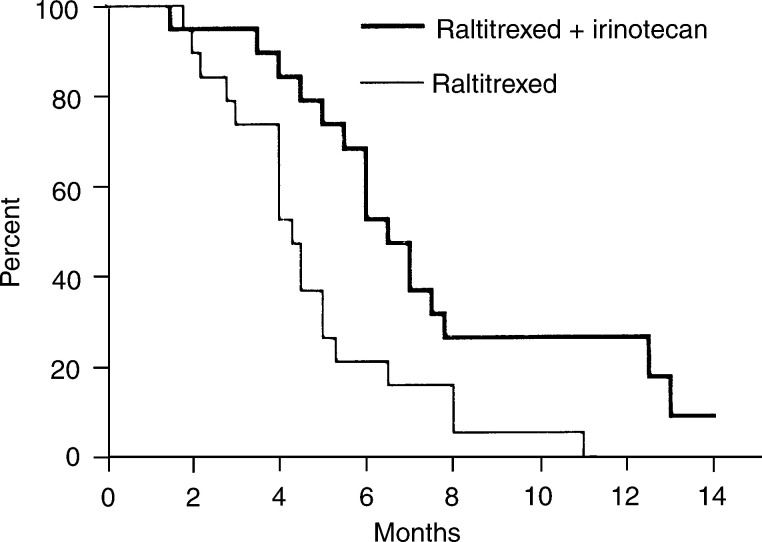
Overall survival.

Disease stabilisation was noted in seven (37%) *vs* six (32%) additional patients in arm A and B for a median duration of 4 (range, 3.2–6) months *vs* 5 (range, 4.2–9) months, respectively. At the time of this analysis, all patients had experienced PD. The median PFS time was 2.5 (range, 1.5–6.0) months for patients in the raltitrexed group and 4.0 (range, 1.5–10.0) months in the combination group. Also in terms of median survival, which was 4.3 (range, 1.8–11.0) months in arm A *vs* 6.5 (range, 1.5–14.0+) months in arm B, a clear benefit was noted in favour of arm B (Figure 1

).

### Clinical benefit response

In total, 28 patients with tumour-related symptoms (pain and/or impaired performance status±weight loss) were considered evaluable for clinical benefit response. In one out of 11 raltitrexed patients suffering from pain at study entry, and in three out of 12 of those in the combination arm, pain intensity and/or analgesic consumption was reduced as compared to baseline values. Three patients (arm A) *vs* five (arm B) were classified as stable in this category. Improvement in pain was accompanied by an improvement in performance status in the patient in the raltitrexed arm, and in two cases in the combination arm. One patient each in the latter arm experienced an improvement in pain without worsening of the performance status or had an improvement in performance status while being rated stable in the pain category. With regard to weight gain, the secondary measure of clinical benefit, there was no patient in either treatment group who had a positive change (>7% increase from baseline). Accordingly, the total number of primarily symptomatic patients experiencing a clinical benefit response was 1 (8%) *vs* 4 (29%) in favour of the combination arm. The median duration to achieve a clinical benefit response was 5 weeks, and its (median) duration was 14 and 17 weeks (range, 12–28 weeks) for raltitrexed-treated and raltitrexed+irinotecan-treated patients, respectively.

### Toxicity

In both treatment arms, toxicity was recorded in all 19 patients. In the raltitrexed arm, a median of three courses was given (range, 2–6; 69 courses were analysed), and in the raltitrexed combination arm, patients received a median of four courses (range, 1–6; 83 courses were analysed). Side effects associated with treatment are listed in Table 2

**Table 2:** Summary of maximum treatment-associated toxicities

	**Number of patients/World Health Organisation toxicity grade (%)**							
	**Raltitrexed**	**Raltitrexed+irinotecan**						
**Toxicity**	**1**	**2**	**3**	**4**	**1**	**2**	**3**	**4**
*Haematological and other laboratory-based toxicity*								
Leukocytopenia	5 (26.3)	2 (10.5)	3 (15.8)	1 (5.3)	6 (31.5)	3 (15.8)	4 (21.0)	1 (5.3)
Neutropenia	3 (15.8)	4 (21.0)	2 (10.5)	1 (5.3)	4 (21.0)	3 (15.8)	3 (15.8)	1 (5.3)
Thrombocytopenia	1 (5.3)	—	—	—	2 (10.5)	1 (5.3)	—	—
Anaemia	7 (36.8)	7 (36.8)	—	—	5 (26.3)	6 (31.5)	—	—
Transaminases	6 (31.5)	3 (15.8)	1 (5.3)	—	4 (21.0)	2 (10.5)	1 (5.3)	—
								
*Symptomatic toxicity*								
Nausea/vomiting	3 (15.8)	4 (21.0)	1 (5.3)	—	3 (15.8)	9 (47.4)	1 (5.3)	—
Stomatitis	1 (5.3)	1 (5.3)	—	—	2 (10.5)	1 (5.3)	—	—
Diarrhoea	4 (21.0)	2 (10.5)	2 (10.5)	—	4 (21.0)	6 (31.5)	2 (10.5)	—
Cholinergic syndrome	—	—	—	—	4 (21.0)	—	—	—
Infection	1 (5.3)	1 (5.3)	—	—	1 (5.3)	1 (5.3)	—	—
Alopecia	—	—	—	—	3 (15.8)	5 (26.3)	—	—
Fatigue	5 (26.3)	2 (10.5)	—	—	5 (26.3)	3 (15.8)	—	—

. Overall, data suggest that both chemotherapeutic drug regimens were fairly well tolerated throughout the study. The most frequently encountered toxicity was myelosuppression, although grade 3/4 leukocytopenia/neutropenia occurred in only four and five patients in arms A and B, respectively. Only two treatment-related minor infections were noted in either group, and grade 3 or 4 thrombocytopenia was not observed. As listed in Table 2, also drug-related symptomatic toxicity was generally mild in both the treatment groups. Gastrointestinal (GI) symptoms, specifically nausea/emesis (42 *vs* 68%) and diarrhoea (42 *vs* 63%) tended to be more frequently observed in the combination arm, although symptoms were severe in only three patients in both groups. The only additional adverse reactions that were more commonly noted in the combination arm were partial alopecia (0 *vs* 42%), and cholinergic syndrome (0 *vs* 21%). Other symptomatic toxicities were equally distributed between the two treatment groups.

In the raltitrexed arm, no patient had a treatment delay, whereas in the raltitrexed+irinotecan combination arm, treatment delays were required in four patients because of intercurrent GI bleeding (*n*=l), biliary stent occlusion (*n*=1), and personal reasons (*n*=2). Dose reductions for adverse reactions according to the study protocol were effected in three patients in the raltitrexed group (for emesis grade 3, and diarrhoea grade 3±leukocytopenia/neutropenia grade 4), and in three cases in the combination group (also for grade 3 GI symptoms±grade 4 haematotoxicity in one). Adverse reactions led to treatment discontinuation in only one patient in the latter group because of continuing nausea/emesis despite dose adjustment.

## DISCUSSION

Owing to the lack of an active anticancer treatment regimen for advanced pancreatic adenocarcinoma, the urgent need for such therapy, and since preclinical data and the results of (meanwhile) two published phase I investigations suggest an encouraging, potentially synergistic activity between raltitrexed and irinotecan (Aschele *et al*, 1998; Ford *et al*, 2000; Stevenson *et al*, 2001), the present randomised phase II study was initiated in patients after gemcitabine failure.

For the combination regimen, feasibility, an encouraging antitumour effectiveness, and a fairly good tolerance were demonstrated. With an IRC-confirmed objective response rate of 16% (a PR was noted in three out of 19 evaluable patients), it was clearly superior to the comparator/control arm with raltitrexed alone, in which no response was obtained. With an additional 32% of patients experiencing SD in the raltitrexed+irinotecan arm (for a median duration of 5 months), this second-line combination regimen resulted in abrogation of progression of this aggressive tumour in about half of our patients. Also the secondary study end points of this trial, median PFS (4.0 months) and OS (6.5 months) seem encouraging. Furthermore, almost one-third of our patients with symptomatic disease (29%) experienced clinically significant and sustained improvements in pain, analgesic consumption and/or Karnofsky performance score. These objective and subjective beneficial effects of raltitrexed+irinotecan were not negated by frequent or severe, clinically relevant treatment-related toxicities. Neutropenia, GI symptoms, asthenia, and transaminitis were commonly noted; however, grade 3 adverse events requiring dose adjustments occurred in only three patients, and there was no treatment-related death.

Comparable therapeutic results in gemcitabine-pretreated patients with advanced pancreatic adenocarcinoma have only occasionally been reported in the past, that is, in a pilot phase II study of irinotecan (Klapdor and Fenner, 2000) and for a rather complex combination regimen consisting of irinotecan, gemcitabine, 5-FU, leucovorin, and cisplatin (Kozuch *et al*, 2001). Other chemotherapeutic drugs, such as paclitaxel (Oettle *et al*, 2000), rubitecan (Stehlin *et al*, 1999), and irofulven (Weitman *et al*, 2001), a unique cytotoxic agent that is related to the mushroom-derived illudins, unfortunately, are only marginally effective or do not seem to hold their promise (D Von Hoff, personal communications).

In conclusion, our data suggest that combined raltitrexed+irinotecan seems to represent an effective salvage regimen in patients with gemcitabine-pretreated metastatic pancreatic adenocarcinoma. In this randomised pick the winner study, we were able to demonstrate a clear superiority compared to the raltitrexed control arm. The choice of the latter drug might be criticised because of the modest activity of the specific thymidilate synthase inhibitor in this disease, even in the front-line setting (Pazdur *et al*, 1996). Based on the results of the two largest, recently published phase III trials with a first-line FU (i.v. short term or protracted venous infusion) arm (Burris *et al*, 1997; Maisey *et al*, 2002); however, alternative use of this antimetabolite (resulting in a response rate of 0–8%, PFS of 1–2.8 months, and OS of 4.4–5.1 months) would probably not have been a better choice and thus have resulted in a different study outcome.

The activity, tolerability, and ease of administration noted in this and other previously reported studies (Ford *et al*, 2000; Stevenson *et al*, 2001), suggest that future trials of irinotecan and raltitrexed in GI cancer patients are warranted. This should certainly include pancreatic cancer, a disease where there is a dire need to improve our therapeutic armentarium, in the front-line as well as in the second-line treatment setting.
